# Supplementary material for: Fusing Sequence and Structural Knowledge by Heterogeneous Models to Accurately and Interpretively Predict Drug–Target Affinity
Source: Molecules. 2023 Dec 8;28(24):8005. doi: 10.3390/molecules28248005 (PMC10745601; doi:10.3390/molecules28248005)
Supplement: Supplementary file 1 [file molecules-28-08005-s001.zip › molecules-2702194-supplementary.pdf]

Supplementary Material

Fusing sequence and structural knowledge by heterogeneous models to accurately and interpretively predict drug-target affinity

Xin Zeng [1], Kai-Yang Zhong [1], Bei Jiang [2] and Yi Li [1*]

1 College of Mathematics and Computer Science, Dali University, Dali 671003, China; xinzeng@dali.edu.cn (X.Z.); zkaiyang2022@163.com (K.-Y.Z.)

2 Yunnan Key Laboratory of Screening and Research on Anti-Pathogenic Plant Resources from Western Yunnan, Dali University, Dali 671000, China; jiangbei@dali.edu.cn

* Correspondence: yili@dali.edu.cn

Detailed summary of the dataset

In line with current competing methods [9–11], we utilized a benchmark dataset from the PDBbind database (2016 version) [36] to train, validate, and test our model. This dataset comprises 13283 high-resolution structures of drug-target complexes sourced from the Protein Data Bank (PDB, https://www.rcsb.org), along with experimental values of DTA, typically represented by pK_d. The training and testing datasets were constructed from the PDBbind database (2016 version), resulting in 12993 and 290 samples, respectively. To ensure the training efficiency of the model, the sequence length of targets was fixed at 2100 amino acids to cover 98.74% of samples (Supplementary Figure S1) of the training dataset. A total of four samples were excluded due to processing issues with the Biopython package [37]. Consequently, the training dataset comprises 12823 samples, while the testing dataset has 289 samples. The maximum length of drug SMILES (Simplified Molecular Input Line Entry System) [20] was chosen as the fixed length. Sequences of targets or drug SMILES shorter than their fixed lengths were zero-padded.

Supplementary Figure S1

A sequence length distribution statistic chart of targets in 2016 version dataset of PDBbind database.

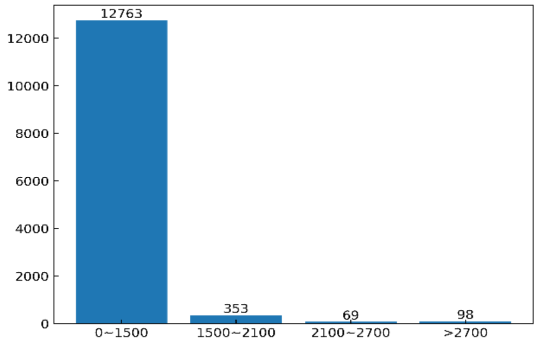

Supplementary Table S1

The physical-chemical properties of amino acids are often also employed for sequence feature of proteins, so we selected 24 physical-chemical properties of amino acids. The data presented in Table S1 is derived from literature [38], which has been compiled from two primary data sources, http://en.wikipedia.org/wiki/Amino acid and http:// en.wikipedia. org/wiki/Proteinogenic amino acid. All properties listed in Table S1 represent the actual numerical values.

24 physical-chemical properties of amino acids in proteins

amino_acid	hydropathy	charge	polarity	volume	count	strength	mjenergy	kf1	kf2	kf3	kf4	kf5	kf6	kf7	kf8	kf9	kf10	rim	surface	turn	alpha	beta	core	disorder
A	1.8	0	0	67	1	0	-2.8455	-1.56	-1.67	-0.97	-0.27	-0.93	-0.78	-0.2	-0.08	0.21	-0.48	0.047	0.065	0.78	1.29	0.9	0.049	0
C	2.5	0	0	86	1	1	-3.782	0.12	-0.89	0.45	-1.05	-0.71	2.41	1.52	-0.69	1.13	1.1	0.015	0.015	0.8	1.11	0.74	0.02	-1
D	-3.5	-1	1	91	1	0	-2.116	0.58	-0.22	-1.58	0.81	-0.92	0.15	-1.52	0.47	0.76	0.7	0.071	0.074	1.41	1.04	0.72	0.051	1
E	-3.5	-1	1	109	1	0	-2.141	-1.45	0.19	-1.61	1.17	-1.31	0.4	0.04	0.38	-0.35	-0.12	0.094	0.089	1	1.44	0.75	0.051	1
F	2.8	0	0	135	1	1	-5.017	-0.21	0.98	-0.36	-1.43	0.22	-0.81	0.67	1.1	1.71	-0.44	0.021	0.029	0.58	1.07	1.32	0.051	-1
G	-0.4	0	0	48	1	0	-2.499	1.46	-1.96	-0.23	-0.16	0.1	-0.11	1.32	2.36	-1.66	0.46	0.071	0.07	1.64	0.56	0.92	0.06	1
H	-3.2	1	1	118	1	0	-2.927	-0.41	0.52	-0.28	0.28	1.61	1.01	-1.85	0.47	1.13	1.63	0.022	0.025	0.69	1.22	1.08	0.034	-1
I	4.5	0	0	124	1	1	-4.641	-0.73	-0.16	1.79	-0.77	-0.54	0.03	-0.83	0.51	0.66	-1.78	0.032	0.035	0.51	0.97	1.45	0.047	-1
K	-3.9	1	1	135	1	0	-1.789	-0.34	0.82	-0.23	1.7	1.54	-1.62	1.15	-0.08	-0.48	0.6	0.105	0.08	0.96	1.23	0.77	0.05	1
L	3.8	0	0	124	1	1	-5.023	-1.04	0	-0.24	-1.1	-0.55	-2.05	0.96	-0.76	0.45	0.93	0.052	0.063	0.59	1.3	1.02	0.078	-1
M	1.9	0	0	124	1	1	-4.1915	-1.4	0.18	-0.42	-0.73	2	1.52	0.26	0.11	-1.27	0.27	0.017	0.016	0.39	1.47	0.97	0.027	1
N	-3.5	0	1	96	1	0	-2.349	1.14	-0.07	-0.12	0.81	0.18	0.37	-0.09	1.23	1.1	-1.73	0.062	0.053	1.28	0.9	0.76	0.058	1
P	-1.6	0	0	90	1	0	-2.443	2.06	-0.33	-1.15	-0.75	0.88	-0.45	0.3	-2.3	0.74	-0.28	0.052	0.054	1.91	0.52	0.64	0.051	1
Q	-3.5	0	1	114	1	0	-2.2505	-0.47	0.24	0.07	1.1	1.1	0.59	0.84	-0.71	-0.03	-2.33	0.053	0.051	0.97	1.27	0.8	0.051	1
R	-4.5	1	1	148	1	0	-2.402	0.22	1.27	1.37	1.87	-1.7	0.46	0.92	-0.39	0.23	0.93	0.068	0.059	0.88	0.96	0.99	0.066	1
S	-0.8	0	1	73	1	0	-2.308	0.81	-1.08	0.16	0.42	-0.21	-0.43	-1.89	-1.15	-0.97	-0.23	0.072	0.071	1.33	0.82	0.95	0.057	1
T	-0.7	0	1	93	1	0	-2.6145	0.26	-0.7	1.21	0.63	-0.1	0.21	0.24	-1.15	-0.56	0.19	0.064	0.065	1.03	0.82	1.21	0.064	0
V	4.2	0	0	105	1	1	-4.093	-0.74	-0.71	2.04	-0.4	0.5	-0.81	-1.07	0.06	-0.46	0.65	0.048	0.048	0.47	0.91	1.49	0.049	-1
W	-0.9	0	0	163	1	1	-4.1375	0.3	2.1	-0.72	-1.57	-1.16	0.57	-0.48	-0.4	-2.3	-0.6	0.007	0.012	0.75	0.99	1.14	0.022	-1
Y	-1.3	0	1	141	1	1	-3.7505	1.38	1.48	0.8	-0.56	0	-0.68	-0.31	1.03	-0.05	0.53	0.032	0.033	1.05	0.72	1.25	0.07	-1

Supplementary Table S2

The physical-chemical properties of atoms in drugs also have a large impact on the prediction performance of S2DTA, so we select 9 physical-chemical properties of atoms.

9 physical-chemical properties of atoms in drugs

Atom Property	Atomic number	Atomic mass	Electronegativity (Pauling)	Electronegativity (Allen)	Atomic radius (empirical)	Atomic radius (calculated)	Atomic radius (vanderWaals)	Lonization Energy (eV)	Number of Outermost Electrons
Carbon(C)	6	12.01	2.55	2.54	70	67	170	11.26	4
Hydrogen(H)	1	1.008	2.20	2.3	25	53	120	-13.60	1
Oxygen(O)	8	15.99	3.44	3.61	60	48	152	13.62	6
Nitrogen(N)	7	14.01	3.04	3.07	65	56	155	14.53	5
Fluorine(F)	9	18.99	3.98	4.19	50	42	147	17.42	7
Sulfur(S)	16	32.06	2.58	2.59	100	88	180	10.36	6
Phosphorus(P)	15	30.97	2.19	2.25	100	98	180	10.49	5
Iodine(I)	53	126.90	2.66	2.36	140	115	198	10.45	7
Chlorine(Cl)	17	35.45	3.16	2.87	100	79	175	12.97	7
Arsenic(As)	33	74.92	2.18	2.21	115	114	185	9.79	5
Selenium(Se)	34	78.96	2.55	2.42	115	103	190	9.75	6
Bromine(Br)	35	79.90	2.96	2.69	115	94	185	11.81	7
Boron(B)	5	10.81	2.04	2.05	85	87	192	8.30	3
Platinum(Pt)	78	195.09	2.28	--(2.28)	135	177	175	8.96	2
Vanadium(V)	23	50.94	1.63	1.53	135	171	--(172)	6.83	5
Iron(Fe)	26	55.85	1.83	1.80	140	156	--(172)	7.90	2
Mercury(Hg)	80	200.59	2.00	--(2.28)	150	171	155	10.44	2
Rhodium(Rh)	45	102.91	2.28	1.56	135	173	--(172)	7.46	2
Magnesium(Mg)	12	24.31	1.31	1.29	150	145	173	7.65	2
Beryllium(Be)	4	9.01	1.57	1.58	105	112	153	9.32	2
Silicon(Si)	14	28.09	1.90	1.92	110	111	210	8.15	4
Ruthenium(Ru)	44	101.07	2.2	1.54	130	178	--(172)	7.36	1
Antimony(Sb)	51	121.75	2.05	1.98	145	133	206	8.61	5
Copper(Cu)	29	63.55	1.90	1.85	135	145	140	7.73	1
Rhenium(Re)	75	186.21	1.9	--(2.28)	135	188	--(172)	7.83	2
Iridium(Ir)	77	192.22	2.2	--(2.28)	135	180	--(172)	8.97	1
Osmium(Os)	76	190.2	2.2	--(2.28)	130	185	--(172)	8.44	2

Note: some property values in Supplementary Table S2 are missing (--), we replace the missing values by averaging all occurrences (data in brackets) of this property.
